# Thank You to Our Peer Reviewers!

**Published:** 2016-09-01

**Authors:** The Editors of JADPRO

In this issue of the *Journal of the Advanced Practitioner in Oncology (JADPRO)*, we would like to acknowledge all of the professionals who completed peer reviews for *JADPRO* in the past year. Looking at the credentials noted in the list below, there can be no doubt that as a group, you represent a staggering depth and breadth of experience and expertise in the hematology/oncology field. You do an amazing job at pointing out ways to enhance the articles under consideration while providing positive feedback and encouragement.

We hope that during the past year we have fully given you our thanks and made our appreciation to you clear. We hope that publishing the names of all of the *JADPRO* peer reviewers will be another step toward giving you the recognition you deserve for your incredible work. We couldn’t do it without you!

If you’d like to become a peer reviewer for *JADPRO*, please don’t hesitate to contact claudine@harborsidepress.com for more information about how to get involved.

—From the Editors of *JADPRO*

**Paula Anastasia, RN, MN, AOCN®**

**Catherine S. Bishop, DNP, NP, AOCNP®**

**Leigh M. Boehmer, PharmD, BCOP**

**Roy Borchardt, PhD, PA-C**

**Tami Borneman, RN, MSN, CMS**

**Harmony J. Bowles, PharmD**

**Jeannine M. Brant, PhD, APRN, AOCN®**

**Dawn Camp-Sorrell, RN, MSN, FNP, AOCN®**

**Kathryn Ciccolini, BSN, RN, OCN, DNC**

**Diane G. Cope, PhD, ARNP, BC, AOCNP®**

**Heather D. Cox, PharmD, BCOP**

**Sheena Daniels, DNP, ARNP, FNP-BC**

**Mary Caroline Deigert, BS, MPAS, PA-C**

**Andrea S. Dickens, PharmD, BCOP**

**Morgane Diven, PharmD, BCOP**

**Beth Eaby-Sandy, MSN, CRNP, OCN®**

**Denice Economou, RN, MN, CNS, AOCN®, CHPN**

**Beth Faiman, PhD, MSN, APN-BC, AOCN®**

**Debra Friedrich, DNP, FNP-BC, CLS, BC-ADM, FNLA, FAANP**

**Elizabeth Gilbert, MS, PA-C**

**Pamela Ginex, EdD, MPH, BSN**

**Erica Glass, CRNP, MS, RN, BSN, AOCNP®**

**Paige M. Goforth, MMS, PA-C**

**Amy Goodrich, MSN, CRNP**

**Carolyn Grande, CRNP, AOCNP®**

**Heather Greene, MSN, FNP, AOCNP**

**Carol Guarneri, RN, MSN, FNP-C, AOCNS®**

**R. Donald Harvey, PharmD, BCOP, FCCP, FHOPA**

**Lindsay Hladnik, PharmD, BCOP**

**Brianna Hoffner, MS, ANP-BC, AOCNP®**

**Megan Holder, MSN, RN, NP-C, AOCNP®**

**Kate D. Jeffers, PharmD, BCOP**

**Patricia Karwan, DNP, APRN-BC**

**Lisa Kottschade, RN, MSN, CNP**

**Kate Kravits, MA, RN, HNB, LPC, NCC, ATR-BC**

**Tracy Krimmel, AOCN®, APRN-BC**

**Sandra E. Kurtin, RN, MS, AOCN®, ANP-C**

**Lydia T. Madsen, PharmD, RN, AOCNS®**

**Steve Malangone, MSN, FNP-C**

**Jasmine Martin, DNP, MSN, APRN**

**Megan Brafford May, PharmD, BCOP**

**Kelley D. Mayden, MSN, FNP, AOCNP®**

**Erin McMenamin, MSN, CRNP, ANP-BC, AOCN®**

**Patrick Medina, PharmD, BCOP**

**Annette Mercurio, MPH, MCHES**

**Marcia Mickle, ACNP, AOCN®**

**Nancy M. Nix, PharmD, BCPS, BCOP**

**Patricia Palmer, RN, MS, AOCNS®**

**Lisa Parks, MS, RN, CNP, ANP-BC**

**Mary Petrofsky, MS, RN, ACNP, AOCNP®**

**Todd Pickard, MMSC, PA-C**

**Robert Pilarski, MS, LGC, MSW**

**Jacqueline Roberts, DNP, FNP-BC, AOCNP®**

**Barbara Rogers, CRNP, MN, AOCN®, ANP-BC**

**Margaret Quinn Rosenzweig, PhD, FNP-BC, AOCNP®**

**Jean Rosiak, DNP, RN, ANP-BC, AOCNP®, CBCN**

**Meghan Routt, MSN, ANP/GNP-BC, AOCNP®**

**Leanne M. Sakamoto, PharmD, BCOP**

**Julie Scott, ANP-BC**

**Gary Shelton, DNP, NP, ANP-BC, AOCNP®, ACHPN**

**Joseph D. Tariman, PhD, ANP-BC**

**Susan Temple, RN, MSN, AOCN®**

**Carol S. Viele, RN, MS, CNS, OCN®**

**Constance Visovsky, PhD, RN, ACNP-BC**

**Wendy H. Vogel, MSN, FNP, AOCNP®**

**Suzanne Walker, CRNP, MSN, AOCN®, BC**

**Elizabeth Susan Waxman, RN, MSN, AOCN®, NP-C**

**Steven H. Wei, MS, MPH, PA-C**

**Rita Wickham, PhD, RN, AOCN®**

**Elaine Wittenberg, PhD**

**Susan Wojcicki, PharmD, BCOP**

**Elizabeth A. Wolf, PA-C**

**James Young, PharmD**

**Jennifer Zadlo, PharmD, BCOP**

**Laura Zitella, MS, RN, ACNP-BC, AOCN®**

